# Developing better drugs for pulmonary sarcoidosis: determining indications for treatment and endpoints to assess therapy based on patient and clinician concerns

**DOI:** 10.12688/f1000research.20696.1

**Published:** 2019-12-30

**Authors:** Marc A Judson

**Affiliations:** 1 Division of Pulmonary and Critical Care Medicine, Department of Medicine, Albany Medical College, Albany, NY, USA

**Keywords:** Sarcoidosis, treatment, endpoints, corticosteroids, fibrosis, quality of life

## Abstract

Pulmonary sarcoidosis involves the deposition of granulomas within the lung. These granulomas may affect lung function and lead to pulmonary symptoms, pulmonary dysfunction, functional impairment, and worsening of quality of life. Corticosteroids are generally highly effective in resolving the granulomatous inflammation of sarcoidosis. However, despite the effectiveness of corticosteroids, many corticosteroid-responsive patients continue to experience significant problems because of the development of fibrosis from previously active or active smoldering granulomatous inflammation, inflammatory effects from sarcoidosis unrelated to granuloma deposition in lung tissue (parasarcoidosis syndromes), and the development of significant corticosteroid-related side effects. For these reasons, the decision to treat pulmonary sarcoidosis and endpoints to measure meaningful outcomes may extend beyond considerations of pulmonary granulomatous inflammation alone. In this article, we propose a conceptual framework to describe the mechanisms by which pulmonary sarcoidosis significantly impacts patients. This conceptual framework suggests that indications for the treatment of pulmonary sarcoidosis and endpoints to assess treatment depend on the specific mechanisms that are causing functional or quality-of-life impairment (or both) in patients with pulmonary sarcoidosis. We believe that these concepts are important to clinicians treating pulmonary sarcoidosis and to clinical researchers designing pulmonary sarcoidosis trials.

## Introduction

Sarcoidosis is a multisystem granulomatous disease of unknown cause. Granulomas consist of conglomerations of inflammatory cells, predominantly lymphocytes and macrophages. In sarcoidosis, these granulomas may deposit in any organ but most commonly develop within the lung, where they are found in more than 90% of sarcoidosis cases ^1^. The decision to treat pulmonary sarcoidosis is complex because (a) the disease may cause neither symptoms nor permanent organ damage ^2^; (b) a small percentage of cases, maybe 10 to 20%, are progressive or cause fibrosis (that may result in permanent lung dysfunction, significant morbidity, and even mortality) or both ^3^; (c) there are no reliable biomarkers to predict whether the natural course of pulmonary sarcoidosis will be benign or lead to severe impairment ^4^; and (d) treatment of pulmonary sarcoidosis with corticosteroids and other therapies is associated with significant toxicity ^5^. These issues force clinicians, clinical researchers, and pharma to carefully select appropriate pulmonary sarcoidosis patients who have the potential to receive impactful benefits from interventions and to select the appropriate methods to assess therapeutic responses. Clinical trial design for pulmonary sarcoidosis has been problematic, and major trials have been criticized as not adequately accounting for the natural history of the disease in order to determine the benefits of study drugs ^6^. This article will explore the indications for treatment of pulmonary sarcoidosis and methods to assess the effects of therapy. This exercise will involve the construction of a proposed conceptual framework outlining how pulmonary sarcoidosis leads to outcomes that are clinically impactful to patients or clinicians or both. This conceptual framework will suggest that patients in pulmonary sarcoidosis trials require partitioning into disease profiles that each require distinct clinical endpoints to optimally assess the effects of therapy.

## Determining treatment indications on the basis of Wells’s law

### Wells’s law

Because sarcoidosis may affect any organ in the body and its severity is highly variable, it is problematic to define specific criteria for treatment. Most sarcoidosis clinicians agree with Athol Wells’s distillation of the treatment indications for sarcoidosis to just two: situations of danger and significant quality of life (QOL) impairment (“Wells’s law”) ^7^.

### Situations of danger from pulmonary sarcoidosis

Sarcoidosis is a systemic disease, and situations of danger are not confined to the lung. Cardiac sarcoidosis may cause sudden death, eye involvement may result in blindness, and neurosarcoidosis may result in coma and permanent neurologic impairment. In the case of pulmonary sarcoidosis, situations of danger develop in a minority of patients and are listed in Table 1. Dangerous situations from pulmonary sarcoidosis are confined almost exclusively to those who develop fibrocystic disease, which constitutes 10 to 20% of these patients ^8, 9^. Interestingly, the treatment of most of these dangerous situations involves therapy other than anti-granulomatous therapy, although most clinicians and sarcoidosis drug trials focus on obliterating the sarcoid granuloma.

**Table 1.** Situations of danger from pulmonary sarcoidosis.

Condition	Relationship to fibrosis	Mechanism	Treatment	Percentage of patients with pulmonary sarcoidosis
Fibrocystic sarcoidosis	Very strong	The fibrosis is the result of granulomatous inflammation in a subset of patients with sarcoidosis	No known direct treatment. Anti- granulomatous therapy may be beneficial in preventing further development of fibrosis.	10–20%
Sarcoidosis-associated pulmonary hypertension	Strong	Predominantly from fibrotic distortion of the pulmonary vasculature. Other mechanisms may predominate in a minority of patients	Pulmonary vasodilators	5%; most are a subset of patients with fibrocystic sarcoidosis
Bronchiectasis, severe airway distortion	Strong	Fibrotic distortion of airways	Enhance mucociliary clearance, intermittent appropriate antibiotics, possibly roflumilast	5–10%; most are a subset of patients with fibrocystic sarcoidosis
Mycetoma	Very strong	Colonization of fungus in devitalized, fibrotic lung	Surgical excision, anti-fungal agents given systemically or injected into mycetoma cavities	1%; most are a subset of patients with fibrocystic sarcoidosis

The immunopathogenesis of fibrosis in sarcoidosis is not well understood. It is not known whether the fibrosis is triggered by profibrotic inflammatory events, by an inherent predisposition toward fibrosis in a subset of patients, or by an exaggerated wound-healing response to uncontrolled, chronic inflammation ^10^. Mechanisms proposed to be involved in the development of sarcoidosis-associated fibrosis include alveolar macrophage-induced fibrosis ^11^, transition from a T helper 1 to T helper 2 signature ^12^, transforming growth factor-beta ^13^, upregulation of profibrotic genes that may “transmit” signals for fibrosis through the blood compartment ^14^, and M2 polarization of alveolar macrophages ^15^.

**Figure 1.**
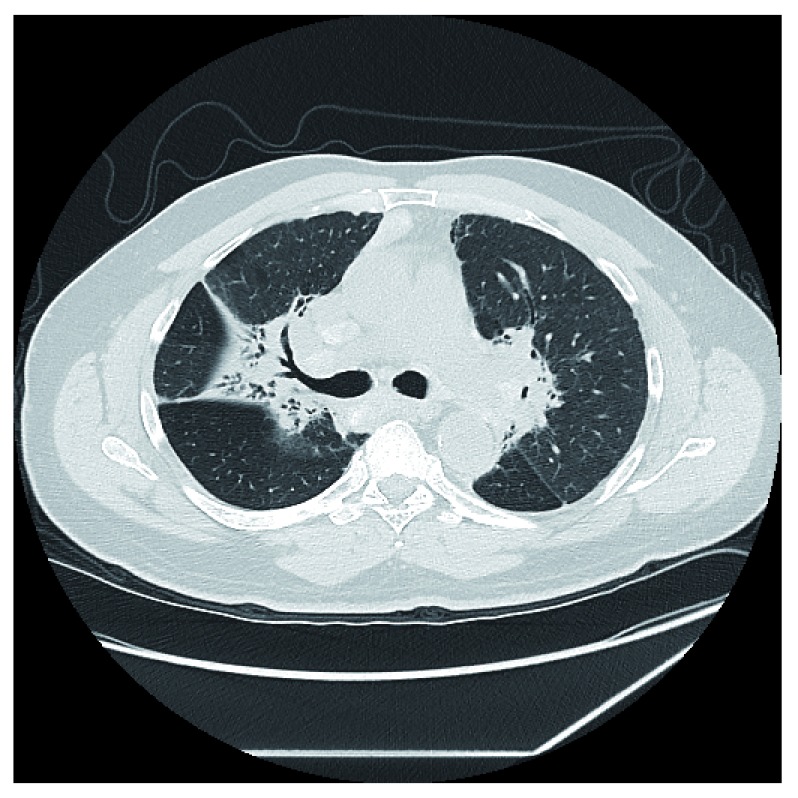
Chest computed tomography image of a patient with fibrotic pulmonary sarcoidosis. Note how the fibrosis is concentrated centrally along the bronchovascular bundles. This is the same location where the granulomas from pulmonary sarcoidosis tend to deposit. This location of fibrosis provides anatomic evidence that the fibrosis in sarcoidosis is a by-product of the granulomatous inflammation of the disease. We confirm that we have obtained permission to use this image from the patient included in this presentation.

It is conjectured that the fibrosis in pulmonary sarcoidosis results from the granulomatous inflammation. This conjecture is supported histologically where the majority of the fibrosis from sarcoidosis develops within or around the granuloma, resulting in a “hyalinized granuloma” ^16^. This conjecture is further supported anatomically through chest imaging studies ^17^ ( Figure 1) as well as analyses of explanted end-stage fibrotic lungs of pulmonary sarcoidosis patients undergoing lung transplantation demonstrating that fibrosis is distributed in a lymphangitic pattern, similar to the usual location of pulmonary granulomas ^16, 18^. Finally, this conjecture is also supported by biomarkers of granulomatous inflammation in that a report of ^18^F-fluorodeoxyglucose ( ^18^F-FDG) positron emission tomography demonstrated significant pulmonary FDG uptake in 22 (85%) of 26 patients with fibrotic pulmonary sarcoidosis ^19^. These data suggest that the granulomatous inflammation of sarcoidosis and the development of fibrosis often go hand in hand ^20^. The amount of fibrosis that develops is variable, from negligent to copious amounts. As previously mentioned, a significant fibrotic response from the granulomatous inflammation of sarcoidosis occurs in, at most, only 20% of cases ^8, 9^. Herein lies a key dilemma in preventing the granulomatous-induced fibrosis of sarcoidosis: since there is no available biomarker to predict which 10 to 20% of patients with pulmonary sarcoidosis will develop significant fibrosis, does the clinician (a) treat all pulmonary sarcoidosis with anti-granulomatous therapy that will prevent the development of fibrosis in 10 to 20% but subject 80% to 90% to potential drug toxicities from corticosteroids and other medications or (b) withhold anti-granulomatous therapy that will result in serious complications in the 10 to 20% who develop fibrosis? Clearly, a reliable biomarker that can accurately predict the development of significant pulmonary fibrosis from sarcoidosis is an unmet need. Effective anti-fibrotic therapy would also be very useful for this form of sarcoidosis, although none is available at present.

### Sarcoidosis-related impairment of quality of life

Because the dangerous situations associated with pulmonary sarcoidosis are not common, the more common of the two indications suggested by Wells’s law to treat pulmonary sarcoidosis is sarcoidosis-induced impairment of QOL. A survey of 1842 European patients with sarcoidosis found that QOL and functionality were judged as the most important disease outcomes ^21^. One could argue that sarcoidosis therapy is used primarily to improve physiology or organ function impaired by granulomatous inflammation or to decrease the granuloma burden , but a critical examination of this issue suggests that this is not the case ^22^. Sarcoidosis-induced granulomatous inflammation may not cause a significant physiologic derangement or result in a significant impairment of QOL ^23^. A common clinical situation where this is the case is with asymptomatic bilateral hilar lymphadenopathy ^24^. Even when the granulomatous inflammation of pulmonary sarcoidosis results in physiologic abnormalities, they are often minor ^8, 23, 25^ and do not invariably lead to appreciable symptoms ^23, 25^. In addition, the correlation between pulmonary dysfunction and pulmonary symptoms is poor in pulmonary sarcoidosis ^26, 27^. Therefore, the presence of granulomatous inflammation or mild physiologic derangements from pulmonary sarcoidosis does not mandate anti-sarcoidosis therapy unless the patient’s QOL is significantly impaired or significant organ dysfunction occurs ^22^.

QOL assessment in pulmonary sarcoidosis is also significantly impacted by the potential toxicity of therapy, particularly corticosteroids. Corticosteroids are the drug of choice for the treatment of pulmonary sarcoidosis ^28, 29^. In most instances, they work quickly and reliably. However, they are associated with a number of potential side effects that may impair QOL. These include weight gain, mood swings/psychological disturbances, diabetes, osteoporosis, hypertension, glaucoma, cataracts, edema, acne, increased skin and vascular fragility, and increased risk of infection. Corticosteroid therapy has been clearly linked to the development of significant toxicities in patients with sarcoidosis ^30^. If the clinician focuses primarily on reducing the granuloma burden or improving physiology, a medication-induced impairment of QOL may be undetected such that the clinician assesses the patient as improved while the patient feels worse off. This scenario may be relatively common, as it has been shown that those receiving corticosteroids at three major university sarcoidosis clinics in the US had statistically and clinically significant reductions in health-related QOL (HRQOL) compared with sarcoidosis patients not receiving corticosteroids ^27^, and those receiving more than 500 mg or prednisone equivalent per year had a statistically and clinically worse HRQOL than those on a lower total yearly dose ^31^.

The assessment of QOL in pulmonary sarcoidosis is problematic. HRQOL refers to the extent that the physical, social, mental/emotional, cognitive, or spiritual domains (or a combination of these) are affected by a medical condition or its treatment ^32^. The HRQOL status of a patient may be assessed informally through unstructured historical questioning. Such an assessment may be thorough and used to make treatment decisions, but in the reality of the current era of patient care where time is compressed, such a detailed evaluation rarely takes place ^22^. For this reason, many have advocated that the assessment of HRQOL in patients with sarcoidosis be assessed by patient-reported outcome measures (PROs), which are patient-administered reports of how they function or feel about their health condition and the effects of therapy ^33, 34^. HRQOL PROs provide a quantitative assessment of QOL and functional status in order to assess potential therapeutic effects. In addition, HRQOL PROs may be performed outside of patient clinic visit time and even serially between visits to assess the effects of therapy. However, there are several limitations of using HRQOL PROs on which to base pulmonary sarcoidosis treatment decisions. First, although some sarcoidosis disease–specific PROs have been developed and validated ^35– 37^, most HRQOL PROs used in patients with sarcoidosis are derived from other disease cohorts. However, an HRQOL PRO derived from non-sarcoidosis cohorts may be used in sarcoidosis provided that there is no plausible rationale that patients with sarcoidosis would assess the trait being measured in a different way than the cohort in which the PRO was validated ^23^. Second, although PROs can reliably assess HRQOL in large clinical cohorts, most PROs have inadequate resolution to be useful in individual patients. PROs developed with modern methodology, such as item response theory (IRT), may have adequate resolution to accurately assess HRQOL in individuals ^38– 40^. Finally, QOL and HRQOL are not synonymous, as many high-priority QOL issues do not pertain to a medical condition or its treatment. It is often problematic to distinguish HRQOL issues from non-health-related QOL issues using HRQOL PROs. Because of these concerns, the role of HRQOL PROs in determining treatment decisions for individual patients with pulmonary sarcoidosis has not been standardized. There is uniform agreement that the clinician should not make treatment decisions in isolation without some insight into the patient’s HRQOL status, preferences, goals, and wishes.

Sarcoidosis may also cause symptoms or organ dysfunction that is not related to tissue deposition of granulomas or the development of fibrosis. These entities are collectively known as parasarcoidosis syndromes ^41^, and they may be responsible for significant QOL and functional impairment. Examples of parasarcoidosis syndromes are erythema nodosum ^42^, small fiber neuropathy ^43^, fatigue syndromes ^44^, pain syndromes ^45^, cognitive decline ^46^, and vitamin D dysregulation ^47^. It has been postulated that these syndromes result from systemic release of mediators associated with the granulomatous inflammation of sarcoidosis ^43, 48^. Although these parasarcoidosis syndromes may not result in appreciable pulmonary symptoms, they may be the result of the granulomatous inflammation from pulmonary sarcoidosis. Parasarcoidosis syndromes may respond to anti-granulomatous therapy ^49, 50^, although the decision to use such therapy must weigh the benefits versus the side effects of treatment in relation to the impact of the parasarcoidosis syndrome ^22^. However, the etiology of parasarcoidosis syndromes is probably more complex than simply a result of granulomatous inflammation, as there may be several non-granulomatous contributions to some of these disorders, such as pain, fatigue, and cognitive impairment syndromes related to the psychological impact of the presence of a chronic disease or decreased function/mobility or both ^51, 52^.

### Conceptual framework of how pulmonary sarcoidosis impacts patients

**Figure 2.**
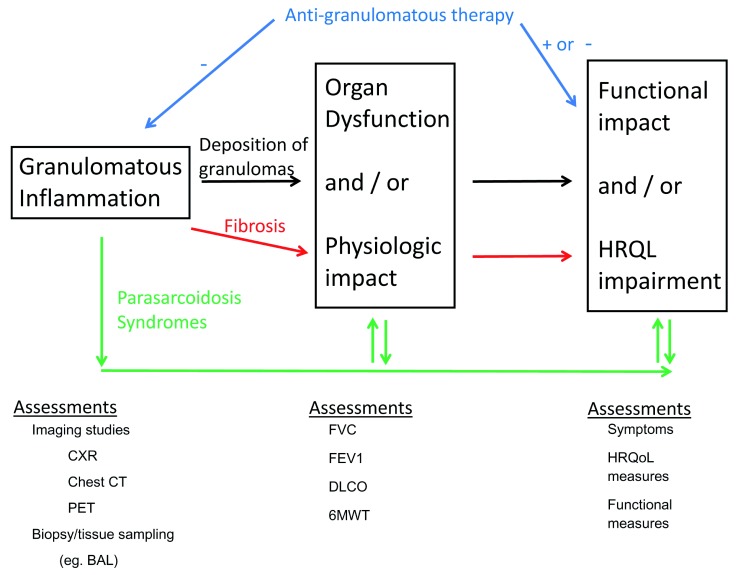
A conceptual framework of how pulmonary sarcoidosis impacts patients. The framework outlines the relationships of granulomatous inflammation, physiologic impairment, fibrosis, parasarcoidosis syndromes, and the impact of anti-sarcoidosis therapy. Assessments refer to methods to assess the processes described within the boxes. Black arrows show the potential clinical progression of pulmonary sarcoidosis directly related to pulmonary deposition of sarcoidosis granulomas. This process is potentially reversible. Red arrows show the potential clinical progression of pulmonary sarcoidosis from pulmonary fibrosis. This process is irreversible. Green arrows show the potential clinical progression of pulmonary sarcoidosis from the development of parasarcoidosis syndromes. These processes may be reversible. Blue arrows show the effects of anti-granulomatous therapy that may reduce granulomatous inflammation (−) and thereby improve health-related quality of life (+). However, the toxicity of anti-granulomatous therapy, particularly corticosteroids, may worsen health-related quality of life (−). 6MWT, 6-minute walk test; BAL, bronchoalveolar lavage; CT, computed tomography; CXR, chest x-ray; DLCO, diffusing capacity of carbon monoxide; FEV1, forced expiratory volume in 1 second; FVC, forced vital capacity; HRQL, health-related quality of life; HRQoL, health-related quality of life; PET, positron emission tomography.

We have proposed a conceptual framework of how pulmonary sarcoidosis impacts patients through the relationships of granulomatous inflammation, physiologic impairment, fibrosis, parasarcoidosis syndromes and the impact of anti-sarcoidosis therapy ( Figure 2). This conceptual framework will serve as the blueprint for the selection of the appropriate patients with pulmonary sarcoidosis for therapy and the assessment of such therapy that will be outlined below. Based on this conceptual framework, the treatment of pulmonary sarcoidosis can be directed against three different aspects of the disease: anti-granulomatous therapy for acute untreated disease, anti-granulomatous therapy for chronically treated disease, and therapy for fibrotic sarcoidosis.

### What the conceptual framework does not measure

This conceptual framework is directed at immunologic and physiologic factors of pulmonary sarcoidosis that impact patient health. It reflects relevant issues concerning the evaluation of these drugs in clinical pulmonary sarcoidosis trials, including the establishment of study entry criteria and clinically relevant endpoints. However, issues concerning the management of patients with pulmonary sarcoidosis extend beyond these immunologic and physiologic factors. For example, psychosocial and socioeconomic concerns are important considerations of therapy. Such concerns include the cost of care, including medication costs, access to care, social support, employment issues, emotional and psychological issues, and ease in maintaining a healthy lifestyle.

## The approach to anti-granulomatous therapy for acute untreated pulmonary sarcoidosis

Acute pulmonary sarcoidosis is a form of the disease that causes worsening pulmonary symptoms and dysfunction over weeks to months and is thought to result from the formation of new granulomas within the lung or growth of existing pulmonary granulomas ^53^. Obviously, anti-granulomatous therapy is a rational approach to the treatment of this condition. As depicted by the black arrows in Figure 2, the sarcoidosis granulomas deposit in involved organs such as the lung and impair normal physiology. These physiologic derangements may lead to functional and QOL impairment. As previously mentioned, the correlations between sarcoidosis-induced granulomatous inflammation and physiologic derangements and between physiologic derangements and QOL impairment are weak.

Obviously, patients with pulmonary sarcoidosis should be considered for anti-granulomatous therapy only if they have evidence of active granulomatous inflammation. Table 2 lists clinical criteria that have been used to determine that granulomatous inflammation is present. These criteria have been used by clinicians to decide when to treat pulmonary sarcoidosis with anti-granulomatous therapy and as entry criteria in pulmonary sarcoidosis trials of anti-granulomatous drugs ^54– 58^. However, as previously mentioned, because the granulomatous inflammation of sarcoidosis may not progress or even spontaneously resolve without therapy and therapy is associated with significant toxicity, treatment of acute pulmonary sarcoidosis also requires that the condition has resulted in a significant impairment of QOL.

**Table 2.** Methods to determine the presence of active pulmonary sarcoidosis.

Method	Reliability in reflecting active pulmonary granulomatous inflammation	Positive features	Negative features
Development of pulmonary symptoms: cough, dyspnea, wheeze, and chest pain	++	Focuses on the pulmonary physiologic impact of the disease	Pulmonary symptoms are not highly sensitive or specific for acute pulmonary sarcoidosis. They may represent alternative pulmonary conditions or permanent fibrotic change from previously active pulmonary sarcoidosis
Serum biomarkers	+	Quantifiable. Directly reflect the total body granuloma burden	Not specific for granulomatous inflammation in the lung
Bronchoalveolar lavage fluid findings (lymphocytosis and elevated CD4/CD8 ratio)	++++	Directly reflects the pulmonary inflammation burden	Cumbersome and relatively invasive
Changes in chest imaging	++	Specific radiographic findings are highly specific for active lung inflammation	Often insensitive to significant change, especially in the case of chest radiographs. Chest CT scans pose a significant radiation risk
Positive FDG uptake on lung PET scan	++++	Specifically reflects active lung inflammation	The threshold for clinical significance of the change in FDG uptake is unknown

CT, computed tomography; FDG, fluorodeoxyglucose; PET, positron emission tomography; +, fair; ++, fair-good: ++++, excellent.

**Figure 3.**
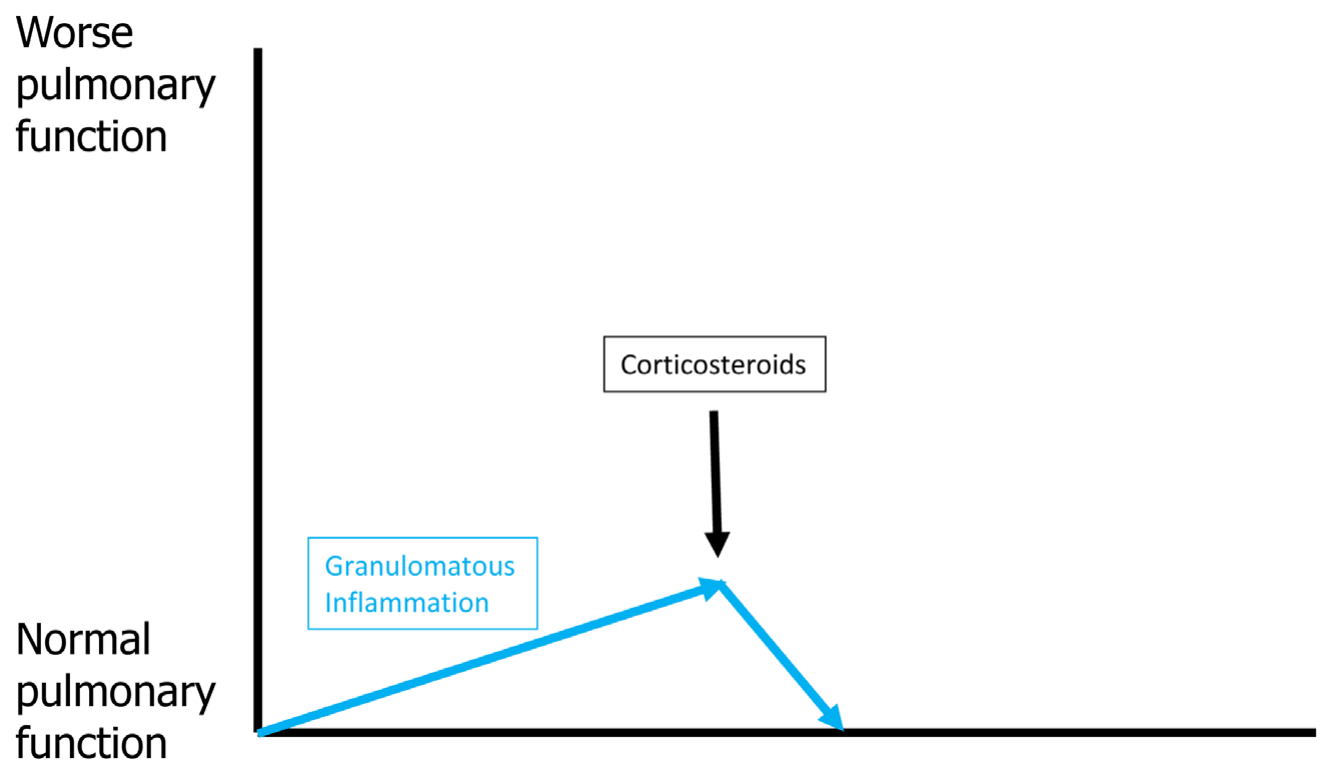
The effect on pulmonary function of corticosteroids for untreated, active pulmonary sarcoidosis. Treatment of the granulomatous inflammation of pulmonary sarcoidosis (blue arrows) with corticosteroids (black arrow) should result in clearance of the deposited granulomas within the lung and return pulmonary function back to normal if no appreciable lung fibrosis has yet developed. It is important to note that, because of potential corticosteroid drug toxicities, such therapy may not resolve quality-of-life issues.

**Figure 4.**
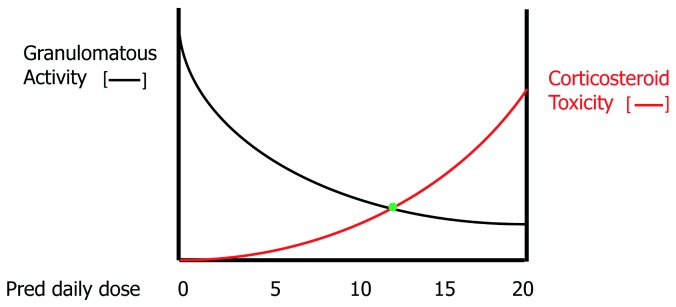
The relationship between corticosteroid dose and pulmonary granulomatous activity (black line) and risk of corticosteroid toxicity (red line) for the treatment of pulmonary sarcoidosis. Increasing corticosteroid doses are more effective in resolving granulomatous inflammation at the cost of a greater risk of corticosteroid toxicity. The clinician attempts to locate a “sweet spot” (green dot) where anti-granulomatous efficacy is significant without appreciably raising the risk of corticosteroid side effects.

Figure 3 shows the effect of corticosteroid therapy on pulmonary function for acute untreated pulmonary sarcoidosis. Corticosteroids are most commonly used as initial treatment for acute untreated pulmonary sarcoidosis because they work almost universally and work more rapidly than other medications. If no fibrosis has developed, effective granulomatous therapy should extinguish all granulomatous inflammation and return lung tissue to its normal state, resulting in complete resolution of physiologic impairment. Although resolution of pulmonary physiologic impairment might resolve QOL and functional impairment, this may not be the case because of the potential for the patient to develop numerous corticosteroid side effects. Figure 4 shows a hypothetical relationship between the efficacy of corticosteroids in resolving the granulomatous inflammation of sarcoidosis and the risks of corticosteroid toxicity. Although high doses of corticosteroids are effective in suppressing granulomatous inflammation, they accomplish this by placing the patient at high risk of corticosteroid side effects. The clinician often attempts to locate a “sweet spot” daily dose of corticosteroids where granulomatous inflammation is adequately suppressed without inducing significant corticosteroid toxicity. Because many of the side effects of corticosteroids (for example, weight gain, osteoporosis, and cataract formation) are cumulative, the duration of corticosteroid therapy may affect the tolerable maximum daily dose of corticosteroids that can be safely used. For this reason, it has been advocated that the daily corticosteroid dose be tapered for pulmonary sarcoidosis cases that have responded to therapy. Although intermittent short courses of corticosteroids may be effective in these instances, acute relapses of pulmonary sarcoidosis are very common such that this treatment approach is rarely satisfactory ^59^.

The endpoints for the treatment of acute untreated pulmonary sarcoidosis should include all three of the following: (a) improvement/resolution of granulomatous inflammation, (b) improvement in pulmonary physiology, and (c) improvement in function status or QOL or both. The rationale for this is explained in Table 3, where it is demonstrated that when any one of these endpoints is not reached, the benefit of treatment of acute untreated pulmonary sarcoidosis is questionable. This does not imply that each clinical pulmonary sarcoidosis trial must incorporate all of these endpoints. For example, if a drug therapy has been previously demonstrated to reliably reduce or eliminate the granulomatous inflammation of sarcoidosis, it may not be necessary to demonstrate this endpoint in a clinical trial, although evidence of granulomatous inflammation should be a requirement for study entry.

**Table 3.** Assessment of clinical responses to an anti-granulomatous intervention for acute pulmonary sarcoidosis.

Granulomatous inflammation	Organ function/ Physiology	HRQL/function	Usefulness of the intervention
+	+	+	Potentially useful
+	−	−	Not clinically useful
+	−	+	Potentially useful, but the mechanism of action may be unclear (for example, corticosteroids causing euphoria)
+	+	−	Not clinically useful
−	+	+	Potentially useful, but the mechanism of action may be unclear (for example, treatment of heart failure or obesity)
−	+	−	Not clinically useful
−	−	+	Potentially useful, but the mechanism of action may be unclear (for example, corticosteroids causing euphoria)
−	−	−	Not clinically useful

HRQL, health-related quality of life; +, present; -, absent.

## The approach to anti-granulomatous therapy for chronically treated pulmonary sarcoidosis

**Figure 5.**
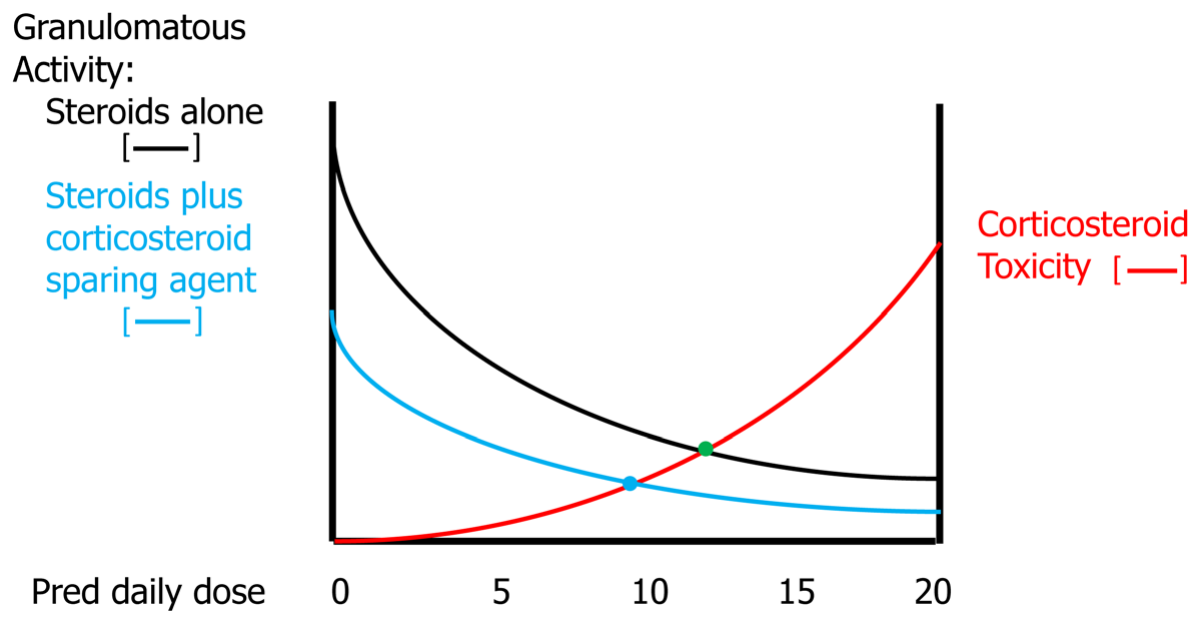
The rationale for adding a corticosteroid-sparing drug for the treatment of pulmonary sarcoidosis. A corticosteroid-sparing drug has some anti-granulomatous activity such that a lower dose of corticosteroids (blue line) is needed to achieve the same level of granulomatous activity as without use of the corticosteroid-sparing medication (black line). This results in lowering the sweet spot for the ideal corticosteroid dose (from the green dot to the blue dot).

Because corticosteroids are highly efficacious for pulmonary sarcoidosis, additional anti-granulomatous agents are often used as corticosteroid-sparing agents in order to lower the corticosteroid dose requirement. Additional agents can also be used to provide further anti-granulomatous therapy on top of corticosteroids, although often the additional benefit of such agents is minimal in pulmonary sarcoidosis patients receiving more than 10 to 15 mg of prednisone per day ^60^. Figure 5 outlines how a corticosteroid-sparing agent can effectively decrease the corticosteroid dose sweet spot in terms of the benefits of corticosteroid therapy versus the risks of corticosteroid toxicity. A majority of 36 sarcoidosis experts believed that a chronic daily maintenance dose of more than 10 mg of prednisone was unacceptable and mandated consideration of corticosteroid-sparing therapy ^61^. For pulmonary sarcoidosis, these corticosteroid-sparing agents include anti-metabolites such as methotrexate ^62, 63^, azathioprine ^64, 65^, and the tumor necrosis factor-alpha (TNF-α) antagonists infliximab ^56^ and adalimumab ^66^. These agents usually are used as second- or third-line agents that are added on to corticosteroid therapy because they either take weeks to months longer than corticosteroids to be effective ^63, 65^ or—in the case of the TNF-α antagonists—are much more expensive than corticosteroids.

**Figure 6.**
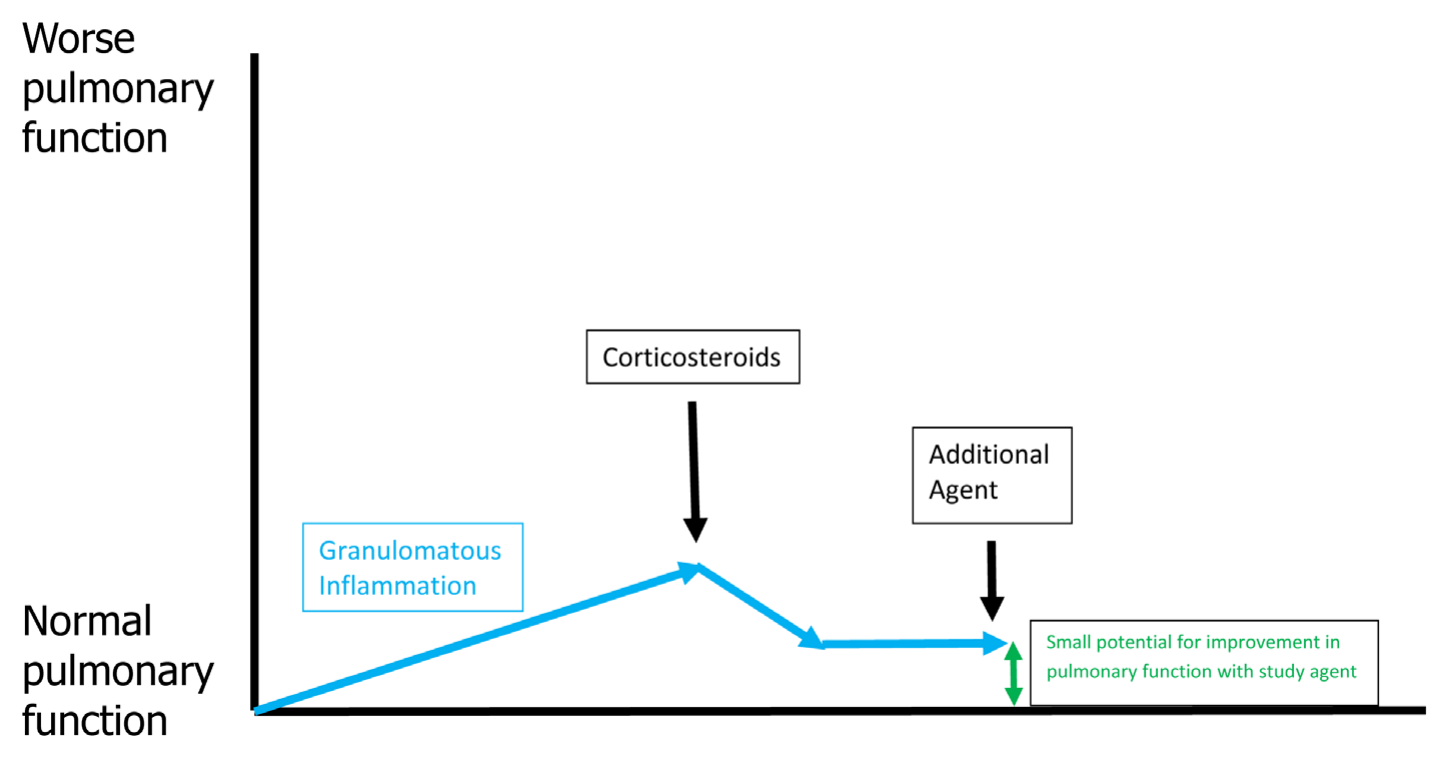
The effect on pulmonary function of adding an additional anti-granulomatous drug to corticosteroid treatment for chronically treated pulmonary sarcoidosis. There is usually minimal residual granulomatous inflammation in patients who receive corticosteroids such that there is a small potential for further significant physiologic improvement (green double arrow). It is important to note that although additional drug therapy may not greatly improve physiology, it may significantly improve quality of life by reducing corticosteroid side effects if addition drugs are corticosteroid-sparing.

Entry criteria for anti-granulomatous therapy trials for patients with chronically treated pulmonary sarcoidosis should include evidence of granulomatous inflammation, physiologic impairment, and functional/QOL impairment at study entry or—if a corticosteroid withdrawal phase of the trial is planned—historical evidence of these abnormalities when the corticosteroid dose is lowered. Figure 6 outlines the effect on pulmonary physiology with the addition of a corticosteroid-sparing anti-granulomatous agent for chronically treated pulmonary sarcoidosis. Because corticosteroids are effective anti-granulomatous agents, there is often relatively little residual granulomatous inflammation that requires additional therapy. The prototypical chronic pulmonary sarcoidosis patient enrolled in a clinical trial is receiving chronic corticosteroid therapy. The patient’s pulmonary function has usually waxed and waned over time, depending on the dose of corticosteroids and other anti-granulomatous drug therapy. The pulmonary function of these patients may decline if their granulomatous inflammation is undertreated, and they may develop permanent reductions in pulmonary function from the development of pulmonary fibrosis. However, this fibrosis rarely causes a rapid decline in physiology, as evidenced by the fact that the placebo group in several trials where all subjects were receiving a maintenance corticosteroid medication in addition to a study drug did not demonstrate a significant reduction in spirometry over a study period of 4 months or more ^55, 56^.

For these reasons, the endpoints of trials for acute untreated pulmonary sarcoidosis are not highly relevant to evaluate anti-granulomatous drugs used in chronically treated pulmonary sarcoidosis patients. There is often little granulomatous inflammation left to treat, and the issue is whether the drug will continue to suppress granulomatous inflammation when the corticosteroid dose is lowered. The same could be said for the physiologic endpoint: the issue is often not whether the additional drug will improve pulmonary physiology but rather whether it will allow the pulmonary physiology to be maintained as the corticosteroid dose is lowered. The QOL endpoint is still highly relevant, especially in terms of a reduction of corticosteroid side effects. Table 4 outlines potential endpoints for a trial of anti-granulomatous therapy for chronically treated pulmonary sarcoidosis.

**Table 4.** Potential endpoints for trial of anti-granulomatous therapy for chronically treated pulmonary sarcoidosis.

Endpoint	Relevance	Positive features	Negative features
Granulomatous inflammation	++	Examines the anti- granulomatous activity of the drug	There is often minimal residual granulomatous activity remaining to treat. It is problematic to define a reduction in granulomatous inflammation that is significant.
Pulmonary physiology	++	Examines the physiologic impact of the drug	Improvement in physiology correlates weakly with functional and quality-of-life improvement. There is often minimal residual physiologic impairment remaining to treat.
Reduction in exacerbations of acute pulmonary sarcoidosis	+++	Often a major concern of patients and clinicians	Acute pulmonary exacerbations of sarcoidosis are often problematic to differentiate from other pulmonary conditions.
Reduction in corticosteroid dose/corticosteroid side effects	++++	Often a major concern of patients and clinicians	A corticosteroid reduction may be successful when the study drug is not active if the underlying granulomatous inflammation of sarcoidosis has lessened or resolved.
Improved functional status/ quality of life	++++	Usually the major concern of patients and clinicians	The improved functional status/quality of life may not be health-related.

++, somewhat relevant; +++, relevant; ++++, highly relevant.

## The approach to therapy for fibrotic pulmonary sarcoidosis

**Figure 7.**
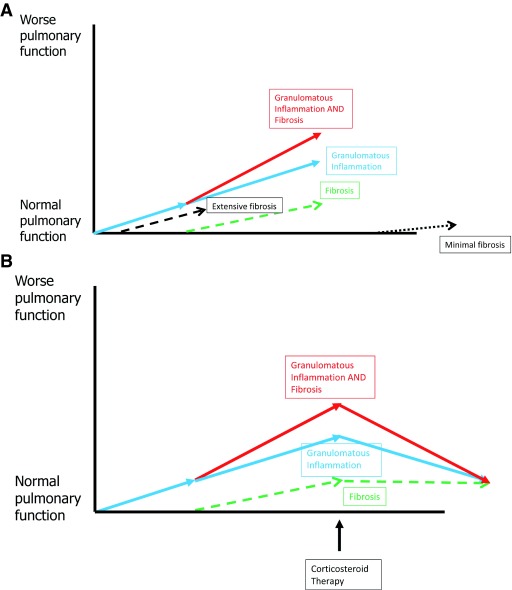
Development and treatment of pulmonary fibrosis in sarcoidosis. **(A)** Development and progression of pulmonary fibrosis in a patient with active pulmonary sarcoidosis. The granulomatous inflammation of sarcoidosis may result in worsening pulmonary symptoms and worsening pulmonary function (blue line). Because the granulomatous inflammation of sarcoidosis is the most common cause of pulmonary fibrosis in sarcoidosis, fibrosis may develop as the granulomatous inflammation continues (green line). This fibrosis contributes to worsening pulmonary symptoms and worsening pulmonary dysfunction (red line). In some patients with pulmonary sarcoidosis, the fibrotic reaction to the granulomatous inflammation may be robust (wide dotted line), causing extensive fibrosis early. Other patients with pulmonary sarcoidosis may have a sluggish fibrotic response to the granulomatous inflammation (short dotted line), and the fibrosis is minimal and requires granulomatous inflammation to be active for a longer period of time. Some patients with pulmonary sarcoidosis may not develop fibrosis at all in response to the granulomatous inflammation. **(B)** The effect of treatment on the granulomatous inflammation and fibrosis associated with pulmonary sarcoidosis. Effective anti-granulomatous therapy will suppress the granulomatous inflammation of sarcoidosis (blue line) and improve the overall pulmonary symptoms and pulmonary function (red line). However, anti-granulomatous therapy will have no effect on the pulmonary symptoms or dysfunction caused by sarcoidosis-related fibrosis (green line). Therefore, successfully treated patients will have their symptomatic and physiologic improvement limited by the degree of pulmonary fibrosis that has developed.

As previously mentioned, it is conjectured that the fibrosis in sarcoidosis is the result of granulomatous inflammation. The relationship of granulomatous inflammation, pulmonary fibrosis, and treatment of pulmonary sarcoidosis is depicted in Figure 7. The amount of fibrosis that develops with pulmonary sarcoidosis is highly variable and is essentially unpredictable in individual patients ( Figure 7A). The physiologic defects that develop from fibrosis in pulmonary sarcoidosis are permanent and will not respond to anti-granulomatous therapy or any other therapies that are currently available ( Figure 7B).

Therapy for fibrotic pulmonary sarcoidosis is currently aimed at either (a) treating the complications of pulmonary fibrosis or (b) preventing the progression of further lung fibrosis. The complications of pulmonary fibrosis are listed in Table 1 and include sarcoidosis-associated pulmonary hypertension ^67, 68^, bronchiectasis ^69, 70^, and pulmonary mycetoma ^71, 72^. The indications for treatment of these conditions as well as the endpoints to monitor these forms of disease are well covered elsewhere ^69, 71, 73– 75^. Anti-granulomatous therapy is rarely useful for sarcoidosis-associated pulmonary hypertension and is often detrimental for bronchiectasis and pulmonary mycetoma as it may cause serious infectious complications. As end-stage pulmonary fibrosis is a disabling and life-threatening condition, lung transplantation should be considered in fibrotic pulmonary sarcoidosis patients with limited chance for improvement and minimal extrapulmonary comorbidities ^76^.

In terms of therapy to prevent the development of further fibrosis, it has already been mentioned that the majority of patients with fibrotic pulmonary sarcoidosis have evidence of granulomatous inflammation ^16, 19^. It is thought that such “smoldering” granulomatous inflammation is the nidus for the development of further fibrosis. Therefore, evidence of granulomatous inflammation would be a prerequisite for instituting anti-granulomatous therapy for fibrotic pulmonary sarcoidosis.

An additional issue when considering treatment of sarcoidosis-associated pulmonary fibrosis is the rapidity of its development. It is thought that fibrosis develops slowly in pulmonary sarcoidosis, such that the cumulative toxicities of long-term anti-sarcoidosis medications, especially corticosteroids, would limit their long-term use. Certainly, evidence of rapid development of pulmonary fibrosis with a concomitant rapid decline in physiology or functional status/QOL would be a strong impetus to consider anti-granulomatous therapy. Because of the toxicity of corticosteroids and other anti-granulomatous therapies, another approach would be to use “downstream” medications targeting the development of fibrosis itself. Such medications include pirfenidone and nintedanib, which have been shown to decelerate the development of lung fibrosis in idiopathic pulmonary fibrosis and other interstitial lung diseases ^77– 79^.

In terms of endpoints for the treatment of fibrotic pulmonary sarcoidosis, improvements in pulmonary function and functional status/QOL from anti-granulomatous therapy may be significant yet are unlikely to be large because improvement in the patient’s pulmonary function is unlikely to be great ^57^. The ablation of granulomatous inflammation may improve QOL by reducing symptoms derived from parasarcoidosis syndromes that may be indirectly derived from granulomatous inflammation (see below). However, other useful endpoints for the treatment of fibrotic pulmonary sarcoidosis may reflect a lack of deterioration of patient status. Table 5 shows potential indications for the treatment of fibrotic pulmonary sarcoidosis, and Table 6 shows potential endpoints of treatment for fibrotic pulmonary sarcoidosis.

**Table 5.** Potential indications for treatment of fibrotic pulmonary sarcoidosis.

Indication for treatment	Rationale	Negative features
The presence of granulomatous inflammation	Treating active granulomatous inflammation in fibrotic pulmonary sarcoidosis improves physiology	Fibrosis in sarcoidosis usually develops slowly such that obliteration of granulomatous inflammation may have a minimal appreciable effect on the development of fibrosis.
Significant impairment in functional status or quality of life from previous pulmonary fibrosis	Usually the major concern of patients and clinicians	No anti-fibrotic therapy that lessens the degree of pulmonary fibrosis is currently available.
Relatively rapid decline in functional status or quality of life impairment from pulmonary fibrosis	Usually the major concern of patients and clinicians	No anti-fibrotic therapy for pulmonary sarcoidosis is currently available.
Development of a condition associated with fibrotic pulmonary sarcoidosis ( Table 1)	These conditions may cause significant impairment of quality of life or dangerous conditions or both	Most of these treatments are for complications of pulmonary fibrosis and do not directly treat the presence or worsening of pulmonary fibrosis.

**Table 6.** Potential endpoints for fibrotic pulmonary sarcoidosis.

Endpoint	Clinical usefulness	Positive features	Negative features
Resolution of granulomatous inflammation	+	Strongly suggests that the profibrotic response has been ablated	Problematic to quantify degree of reduction of granulomatous inflammation
Time to clinical worsening of pulmonary function	++	Suggests that intervention is decelerating the physiologic decline	Correlation of physiology to functional status and quality of life is poor; development of pulmonary fibrosis is slow such that it may take a long time to detect significant differences
Time to clinical worsening of functional status or quality of life	+++	Usually the major concern of patients and clinicians	Development of pulmonary fibrosis is slow such that it may take a long time to detect significant change; problematic to distinguish health-related from health-unrelated quality-of-life effects
Reduction in corticosteroid dose/corticosteroid side effects	+++	Often a major concern of patients and clinicians	A corticosteroid reduction may be successful when a drug is ineffective if the underlying granulomatous inflammation of sarcoidosis has lessened or resolved

+, poor to somewhat useful; ++, useful; +++, very useful.

## Summary

The physiologic impairment resulting from the granulomatous inflammation of pulmonary sarcoidosis is highly variable. Even more variable is the effect of these physiologic derangements on the QOL of the patient with pulmonary sarcoidosis. This granulomatous inflammation can also adversely affect the QOL of the patient with sarcoidosis by systemic mechanisms other than direct deposition of granulomas into specific organs (“parasarcoidosis syndromes”). Additionally, this granulomatous inflammation may lead to the development of pulmonary fibrosis that causes irreversible pulmonary dysfunction and, potentially, disabling and life-threatening situations. Finally, anti-granulomatous therapy not only may improve the QOL of the patient with pulmonary sarcoidosis by effectively treating the disease but also may worsen it by causing serious drug side effects. We have proposed a conceptual framework to outline how these many factors interact to cause physiologic and QOL impairments in pulmonary sarcoidosis. We believe that this conceptual framework may be useful in constructing a clinical drug trial for pulmonary sarcoidosis that reflects the concerns of patients and clinicians.

Because of numerous aspects of pulmonary sarcoidosis and its therapy, it is not surprising that developing treatment indications and endpoints for the therapy of the disease is challenging. We have made a case for partitioning patients into three treatment groups when considering indications for therapy as well as therapy endpoints: untreated patients with active granulomatous inflammation, patients with chronically treated disease, and those with significant lung fibrosis. It is hoped that this proposed patient partitioning will stimulate debate and constructive criticism that will eventually lead to therapy for pulmonary sarcoidosis that is better directed in treating the true concerns of patients.
